# Synthesis and biological characterisation of ^18^F-SIG343 and ^18^F-SIG353, novel and high selectivity σ_2_ radiotracers, for tumour imaging properties

**DOI:** 10.1186/2191-219X-3-80

**Published:** 2013-12-11

**Authors:** Vu H Nguyen, Tien Pham, Chris Fookes, Paula Berghofer, Ivan Greguric, Andrew Arthur, Filomena Mattner, Gita Rahardjo, Emma Davis, Nicholas Howell, Marie-Claude Gregoire, Andrew Katsifis, Rachael Shepherd

**Affiliations:** 1 LifeSciences, ANSTO, Locked Bag 2001, Kirrawee, New South Wales 2232, Australia; 2 Department of PET & Nuclear Medicine, Royal Prince Alfred Hospital, Camperdown, New South Wales 2050, Australia

**Keywords:** ^18^F, Sigma receptors, Phthalimide, A375 cell line, PET imaging, σ_2_ radiotracers

## Abstract

**Background:**

Sigma2 (σ_2_) receptors are highly expressed in cancer cell lines and in tumours. Two novel selective ^18^F-phthalimido σ_2_ ligands, ^18^F-SIG343 and ^18^F-SIG353, were prepared and characterised for their potential tumour imaging properties.

**Methods:**

Preparation of ^18^F-SIG343 and ^18^F-SIG353 was achieved via nucleophilic substitution of their respective nitro precursors. *In vitro* studies including radioreceptor binding assays in the rat brain membrane and cell uptake studies in the A375 cell line were performed. *In vivo* studies were carried out in mice bearing A375 tumours including positron emission tomography (PET) imaging, biodistribution, blocking and metabolite studies.

**Results:**

*In vitro* studies showed that SIG343 and SIG353 displayed excellent affinity and selectivity for σ_2_ receptors (Ki(σ_2_) = 8 and 3 nM, σ_2_:σ_1_ = 200- and 110-fold, respectively). The σ_2_ selectivity of ^18^F-SIG343 was further confirmed by blocking studies in A375 cells, however, not noted for ^18^F-SIG353. Biodistribution studies showed that both radiotracers had similar characteristics including moderately high tumour uptake (4%ID/g to 5%ID/g); low bone uptake (3%ID/g to 4%ID/g); and high tumour-to-muscle uptake ratios (four- to sevenfold) up to 120 min. Although radiotracer uptake in organs known to express σ receptors was significantly blocked by pre-injection of competing σ ligands, the blocking effect was not observed in the tumour. PET imaging studies indicated major radioactive localisation in the chest cavity for both ligands, with approximately 1%ID/g uptake in the tumour at 120 min. Metabolite studies showed that the original radiotracers remained unchanged 65% to 80% in the tumour up to 120 min.

**Conclusions:**

The lead ligands showed promising *in vitro* and *in vivo* characteristics. However, PET imaging indicated low tumour-to-background ratios. Furthermore, we were unable to demonstrate that uptake in the A375 tumour was σ_2_-specific. ^18^F-SIG343 and ^18^F-SIG343 do not display ideal properties for imaging the σ_2_ receptor in the A375 tumour model. However, since the radiotracers show promising *in vitro* and *in vivo* characteristics, longer scans using appropriate half-life isotopes and alternative tumour models will be carried out in future studies to fully validate the imaging characteristics of these radiotracers.

## Background

Since its discovery [1], sigma (σ) receptor nomenclature had undergone considerable changes and has now been generally accepted as a distinct biochemical entity whose exact functions still remain elusive. There are at least two subtypes of σ receptors, σ_1_ and σ_2_, which are distinguished from each other by their ligand selectivity, molecular size and postulated pharmacological profile [2-4].

The σ receptors have been characterised in several species, including human, and have been found unevenly distributed in the brain [5,6], the endocrine system including the liver, spleen, and pancreas [4,7] and in other peripheral organs such as the testis, ovary, heart and kidney [8-10]. Sigma receptor subtypes were also found to be both region- and species-specific. For instance, σ_1_ receptors were found in higher concentration compared to σ_2_ receptors in the brain [5,6] and in the liver of guinea pig compared to other species [4].

The σ_1_ gene has been cloned from a variety of mammalian tissue sources [11-15] and shares no sequence homology to any other cloned mammalian protein. The σ_1_ receptor was recently identified as a receptor chaperone in the endoplasmic reticulum (ER) and is involved in ER-mitochondrial Ca^2+^ signalling and cell survival [16,17]. To date, the structure of σ_2_ receptor is not known, whose gene has also not been purified, sequenced or cloned. As a result, the exact role that this receptor has in tumour and normal cell proliferation is currently unknown. However, recent data have implicated that σ_2_ binding sites is likely to be the progesterone receptor membrane component 1 (PMRMC1) or within PMRMC1 complex which shares homology with cytochrome *b*_5_, a heme-binding protein that activates cytochrome P450 proteins [18]. This finding suggested an involvement of σ_2_ receptors in progesterone signalling and lipid and drug metabolism [19], and is in fact in agreement with the hypothesis previously postulated by a number of original reports [12,20-25]. However, the finding remains to be clarified due to the discrepancies in molecular weights, opposite responses to PMRCM1 antibody or σ_2_ receptor antagonists and binding to P450 enzymes of PMRCM1 and σ_2_ receptors [26].

Overexpression of σ receptors in a variety of human tumour cells and tissues such as pancreatic, breast, bladder, melanoma, colon and brain [27-32] has been reported. Interestingly, σ_2_ expression was found to be higher than σ_1_ expression in certain cell lines [27,33,34]. Several studies have shown that σ_2_ receptor expression is a biomarker for the proliferative status of tumour cells and solid tumours [32,35,36], and are 8 to 10 times overexpressed in proliferative cells compared to quiescent cells [32,35]. The PGRMC1 had been reported to be more abundant in several tumours and/or tumour cell lines than in healthy control tissues including the lung [37], breast, colon and thyroid [38] cancer cell lines. Taken collectively, these findings make the σ_2_ receptor an attractive molecular target for the development of new radiotracers not only for tumour detection and assessment of proliferative status, but also for the treatment of cancer using radiolabelled high-affinity/selectivity σ_2_ ligands.

Substantial evidence also demonstrates anti-proliferative and pro-apoptotic activity of σ_2_ agonists in tumour cells [32,35]. Sigma ligands with high affinity, but no selectivity for σ_2_ receptors, have been reported since the 1990s. Over the years, the search for σ_2_ selective ligands has led to the identification of a number compounds having modest to high selectivity for σ_2_ receptors. To date, the majority of σ radioligands used for positron emission tomography (PET) and single-photon emission computed tomography (SPECT) imaging in cancer have either been non-subtype selective or σ_1_ selective [39]. Ligands with some selectivity for σ_2_ receptors have also been reported; however, they are presenting a variety of drawbacks (as summarised in Table 1) including non-specificity (i.e. cross reactivity for other neuroreceptors), low tumour-to-background contrast, short biological half-lives or radiolabelling shortfalls. ^18^F-labelled radiotracers have been implicated to be superior compared to other PET radionuclide-labelled tracers in providing higher quality PET images (higher energy), fewer time constraints (adequate half-life) and in permitting longer scan sessions of higher tumour/normal tissue ratios [40]. Therefore, the need still remains for the development of ^18^F-labelled σ_2_ selective radioligands with optimal tumour uptake and rapid washout from non-target tissues that are suitable for PET or SPECT imaging.

**Table 1:** Overall review of reported selective σ2 ligands for PET and SPECT

**Ligands**	**Radiolabel**	***K***_**i**_^**a **^**or *IC***_**50**_^**b **^**(nM)**		**Comments**	**References**
		**σ**_**1**_	**σ**_**2**_		
	^125^I	554^a^	1.0^a^	Tumour-to-muscle ratio = 6 (4 h, EMT-6 tumoured mice)	[41]
	^76^Br	5484^a^	12^a^	Tumour-to-muscle ratio = 2 (2 h, EMT-6 tumoured mice)	[42]
	^76^Br	12900^a^	8.2^a^	Tumour-to-muscle ratio = 8 (2 h, EMT-6 tumoured mice)	[42]
	^11^C	3078^a^	10^a^	Tumour-to-muscle ratio = 3 (1 h, EMT-6 tumoured mice)	[43]
	^18^F	330^a^	6.9^a^	Tumour-to-muscle ratio = 3 (2 h, EMT-6 tumoured mice)	[40]
	^18^F	2150^a,c^	0.26^a,c^	Tumour-to-muscle ratio = 8 (2 h, EMT-6 tumoured mice)	[40]
	^125^I, ^18^F	2.8^a,d^		Tumour-to-muscle ratio = 8 (2 h, Line 66 breast tumoured mice)	[44]
	^18^F	1711^a^	0.8^a^	Tumour-to-muscle ratio = 2 (2 h, EMT-6 tumoured mice)	[45]
	^18^F	6^b^	2^b^	Brain-to-muscle ratio = 3 (2 h, naïve mice)	[45]

^a^*K*_i_, determined by ANSTO LifeSciences; ^b^*IC*_50_, determined by Caliper LifeSciences; ^c^subtype affinity and selectivity performed in EMT-6 cell line; ^d^affinity performed in line 66 cell line, selectivity not determined.

Recently, to address the shortfalls of previously reported σ_2_ ligands and improve the imaging properties of new σ_2_ ligands, our laboratory has developed a new class of phthalimido σ_2_ ligands with high affinity and selectivity for σ_2_ receptors. The *in vitro* characterisation and structure-affinity analysis of these phthalimido compounds indicated that σ_2_ affinity is significantly enhanced by the phthalimido ring. Functionalising with halogens, such as fluorine, bromine or iodine on the phthalimido ring, would even further increase this σ_2_ affinity. Using this approach has identified two lead PET compounds, ^18^F-SIG343 and ^18^F-SIG353. In the current study, our aim was to explore the tumour imaging potential of the two ligands through further *in vitro* and *in vivo* investigation in mice bearing the A375 human amelanotic melanoma, a cell line that had been reported to express approximately 100 times of σ_2_ receptors higher than its σ_1_ receptor counterpart.

## Methods

### General

All reagents and solvents used were obtained from commercially available sources and used with no further purification. 4-Fluorophthalic anhydride was purchased from Alfa Aesar (Ward Hill, MA, USA) while 4-nitrophthalic anhydride was from Frinton Laboratories Inc. (Hainesport, NJ, USA). Nuclear magnetic resonance (NMR) spectra were performed on a Bruker Avance DPX 400 (Bruker Corporation, Billerica, MA, USA) operating at 400 MHz for ^1^H NMR spectra and 100 MHz for ^13^C NMR spectra. ^18^F-HF was produced on a GE PET trace via the ^18^O(*p*, *n*)^18^F nuclear reaction (RPA Hospital, Camperdown, NSW, Australia). Haloperidol was obtained from Tocris Bioscience (Bristol, UK), (+)-pentazocine from Research Biochemicals Incorporated (Natick, MA, USA) and σ ligands synthesised in house at LifeSciences, ANSTO (Lucas Heights, NSW, Australia). For cell uptake studies, drugs were dissolved in PBS buffer or in saline for injection for the animal studies, with the help of a few drops of 0.5% acetic acid. Semi-preparative high-performance liquid chromatography (HPLC) purification was performed with a Waters 600 HPLC controller (Waters Company, Milford, MA, USA) and pump, an in-line UV detector (Waters 486, 254 nm) and a single sodium diode crystal flow radioactivity detector (Carrol & Ramsey Associates, Berkeley, CA, USA) using a Pheonomenex Bondclone (Lane Cove, New South Wales, Australia) (C18, 10 μm, 300 × 7.8 mm) eluting at 3 mL/min with 30% MeCN/70% water containing 0.1% TFA.

#### ***Purity analysis and specific activity***

Purity analysis and specific activity of ^18^F-SIG343 and ^18^F-SIG353 were performed on a Varian 9002 pump (Varian Medical Systems, Palo Alto, CA, USA), a linear UV-VIS detector (λ = 221 nm) in series with an Ortec ACE Mate Scint 925 γ-detector (Ortec, South Illinois Ave., Oak Ridge, TN, USA) on a Phenomenex Synergi Max-RP (C12, 4 μm, 250 × 4.6 mm) eluting at 1 mL/min with 40% MeCN/60% ammonium acetate (0.1 M) as the mobile phase. The identity of the labelled compounds was confirmed by co-injection with the authentic compounds on HPLC. For specific activity calculations, the radioactivity of the injected product for the radiochemical analysis was measured with a Capintec R15C dose calibrator (Capintec, Inc., Ramsey, NJ, USA), while the mass of SIG343 and SIG353 was determined by comparing the area of the UV absorbance peak corresponding to the carrier product with a calibrated standard curve relating its mass to UV absorbance.

#### ***Lipophilicity***

The lipophilicity of SIG343 and SIG353 were assessed using RP-HPLC by determining the log*P*_7.5_ value using literature procedures [46]. Samples, dissolved in methanol, were analysed using a C18 column (RP C18, Xterra, 5 μm, 4.6 × 150 mm) and a mobile phase consisting of MeOH and phosphate buffer (0.1 M, pH 7.5) compounds were estimated by a comparison of its retention time to that of standards of known log *P* values.

### Chemistry

#### ***Synthesis of SIG343 and SIG353***

##### 2-(4-(6,7-Dimethoxy-3,4-dihydroisoquinolin-2(1H)-yl)butyl)-5-fluoroisoindoline-1,3-dione, *SIG343*

A mixture of 4-(6,7-dimethoxy-3,4-dihydroisoquinolin-2(1H)-yl) butan-1-amine (132 mg, 0.5 mmol) [47], 4-fluorophthalic anhydride (83 mg, 0.5 mmol) and p-xylene (3 mL) was stirred and gently boiled under a stream of nitrogen. As the xylene evaporated, more was added to maintain the volume. Within a few minutes, a viscous pale yellow gum had formed, but this redissolved slowly, disappearing completely after 1.25 h to form a pale yellow solution. Heating was continued for a total of 2 h (approximately 3 mL of additional xylene required), then the hot solution was treated with charcoal, filtered through celite and evaporated. The crystalline residue was recrystallised from 95% ethanol to give 177 mg (85.9%) of large colourless plates. ^1^H NMR (CDCl_3_) δ 1.62 (m, 2H), 1.76 (m, 2H), 2.52 (t, *J* = 7.5 Hz, 2H), 2.68 (t, *J* = 5.8 Hz, 2H), 2.79 (t, *J* = 5.7 Hz, 2H), 3.52 (s, 2H), 3.72 (t, *J* = 7.0 Hz, 2H), 3.82 (s, 3H), 3.83 (s, 3H), 6.50 (s, 1H), 6.57 (s, 1H), 7.36 (ddd, *J* = 8.8, 8.2, 2.4 Hz, 1H), 7.50 (dd, *J* = 7.0, 2.1 Hz, 1H), 7.83 (dd, *J* = 8.2, 4.5 Hz, 1H). ^13^C NMR (CDCl_3_) δ 24.5, 26.5, 28.6, 38.1, 51.0, 55.7, 55.8, 55.9, 57.6, 109.5, 111.2 (d, *J* = 24.5 Hz), 111.5, 121.0 (d, *J* = 23.0 Hz), 125.6 (d, *J* = 9.6 Hz), 126.1, 126.6, 127.8 (d, *J* = 2.3 Hz), 135.1 (d, *J* = 9.2 Hz), 147.2, 147.5, 166.4 (d, *J* = 230.8 Hz), 167.1 (d, *J* = 3.0 Hz), 167.8. Anal. C H N (C_23_H_25_FN_2_O_4_); theoretical C, 66.98; H, 6.11; N, 6.79; found C, 67.07; H, 6.16; N, 6.73.

##### 2-(5-(6,7-Dimethoxy-3,4-dihydroisoquinolin-2(1H)-yl)pentyl)-5-fluoroisoindoline-1,3-dione, *SIG353*

4-Fluorophthalic anhydride and 5-(6,7-dimethoxy-3,4-dihydroisoquinolin-2(1H)-yl)pentan-1-amine were treated under the same reaction conditions for the synthesis of SIG343 to give the title compound as colourless crystals. ^1^H NMR (CDCl_3_) δ 1.41 (m, 2H) 1.71 (m, 4H), 2.60 (m, 2H), 2.84 (m, 4H), 3.66 (s, 2H), 3.68 (t, *J* = 7.1 Hz, 2H), 3.82 (s, 3H), 3.83 (s, 3H), 6.51 (s, 1H), 6.57 (s, 1H), 7.36 (ddd, *J* = 8.8, 8.2, 2.4 Hz, 1H), 7.50 (dd, *J* = 7.0, 2.4 Hz, 1H), 7.83 (dd, *J* = 8.2, 4.7 Hz, 1H).

#### ***Synthesis of the radiolabelling precursors***

##### 2-(4-(6,7-Dimethoxy-3,4-dihydroisoquinolin-2(1H)-yl)butyl)-5-nitroisoindoline-1,3-dione (*1*)

4-Nitrophthalic anhydride and 4-(6,7-dimethoxy-3,4-dihydroisoquinolin-2(1H)-yl)butan-1-amine were treated under the same reaction conditions for the synthesis of SIG343 to give the title compound as bright orange crystals. ^1^H NMR (CDCl_3_) δ 1.64 (m, 2H), 1.81 (m, 2H), 2.53 (t, *J* = 7.5 Hz, 2H), 2.68 (t, *J* = 5.8 Hz, 2H), 2.79 (t, *J* = 5.8 Hz, 2H), 3.51 (s, 2H), 3.79 (t, *J* = 7.2 Hz, 2H), 3.82 (s, 3H), 3.83 (s, 3H), 6.48 (s, 1H), 6.56 (s, 1H), 8.01 (d, *J* = 7.9 Hz, 1H), 8.58 (dd, *J* = 7.9, 2.4 Hz, 1H), 8.64 (d, *J* = 2.4 Hz, 1H). Anal. C H N (C_23_H_25_N_3_O_6_); theoretical C, 62.86; H, 5.73; N, 9.56; found C, 63.03; H, 5.64; N, 9.44.

##### 2-(5-(6,7-Dimethoxy-3,4-dihydroisoquinolin-2(1H)-yl)pentyl)-5-nitroisoindoline-1,3-dione (*2*)

4-Nitrophthalic anhydride and 5-(6,7-dimethoxy-3,4-dihydroisoquinolin-2(1H)-yl) pentan-1-amine were treated under the same reaction conditions for the synthesis of SIG343 to give the title compound as bright orange crystals. ^1^H NMR (d6-DMSO) δ 1.95 (m, 2H), 2.14 (m, 2H), 2.27 (m, 2H), 2.98 (t, *J* = 7.0 Hz, 2H), 3.13 (t, *J* = 5.7 Hz, 2H), 3.22 (t, *J* = 5.7 Hz, 2H), 3.55 (s, 2H), 3.69 (t, *J* = 7.0 Hz, 2H), 4.29 (s, 6H), 7.11 (s, 1H), 7.14 (s, 1H), 8.53 (d, *J* = 7.9 Hz, 1H), 9.04 (d, *J* = 1.8 Hz, 1H), 9.09 (dd, *J* = 7.9, 1.8 Hz, 1H).

### Radiochemistry

An aqueous ^18^F-fluoride solution (^18^F-HF, 6 to 8 GBq) was added to a 10-mL vial containing anhydrous acetonitrile (1 mL), Kryptofix 2.2.2 (2.0 mg; Sigma-Aldrich Corporation, St. Louis, MO, USA) and K_2_CO_3_ (0.7 mg). The solvent was evaporated under a stream of nitrogen at 100°C under vacuum. This azeotropic drying was repeated twice by further addition of anhydrous acetonitrile (2 × 1 mL). The nitro precursors (***1***) or (***2***) (2 mg) was dissolved in DMF (0.5 mL) and added to the dried K222.K_2_CO_3_.K^18^F complex. The reaction was stirred and heated at 150°C for 5 min before the reaction mixture was diluted with mobile phase (500 μL) and purified by semi-preparative reverse-phase chromatography. The peak with the retention time corresponding to SIG343 (16 min) or SIG353 (21 min) was collected and diluted with water (10 mL) and then trapped on a Waters C18 Light Sep Pak^®^. The trapped radiotracer was eluted off the cartridge with ethanol (1 mL). The ethanol was concentrated *in vacuo* and diluted with saline for *in vivo* studies while it was diluted with PBS (pH 7.2) for cell studies.

### *In vitro* studies

#### ***Radioreceptor binding assays***

The test compounds were solubilised in DMSO and diluted in 50 mmol/L Tris-HCl (pH 8.0). Membrane homogenates were prepared from male Sprague-Dawley rat brains as previously described [48,49]. The binding of σ ligands to σ_1_ and σ_2_ receptors was determined according to literature methods with minor modification [50]. Briefly, the percentage of inhibition was determined by incubating, in triplicate, aliquots of diluted rat brain membrane (300 μg of protein) with 10^-11^ to 10^-5^ mol/L concentrations of the studied drugs in 50 mmol/L Tris-HCl (pH 8.0) with ^3^H-(+)-pentazocine (3 nmol/L) at 37°C for 2.5 h for σ_1_ receptors or with ^3^H-DTG (10 nmol/L) and (+)-pentazocine (1 μmol/L) at 25°C for 1.5 h for σ_2_ receptors. In both assays, non-specific binding was determined in the presence of haloperidol (10 μmol/L). After incubation, the reaction was terminated by rapid filtration using a Brandel 48-well cell harvester (Brandel, Gaithersburg, MD, USA) over Whatman GF/B glass fibre filters that were soaked in a solution of 0.5% polyethyleneimine at 4°C for at least 2 h before use. Filters were washed three times with 5 mL of ice-cold wash buffer (50 mmol/L Tris HCl, pH 7.4). The filters were collected and the amount of bound radioactivity was measured using a β-scintillation counter (Tri-Carb 2100TR, Packard Instrument Co., Downers Grove, IL, USA). The percentage inhibition of the studied drugs, at concentrations of 10^-5^ and 10^-7^ mol/L, was also determined for the cross activity at a number of other neuroreceptors (Caliper LifeSciences, MA, USA). The *IC*_50_ values were then converted to apparent *K*_i_ values using the Cheng-Prusoff equation and radioligand *K*_d_ values [51].

#### ***Cell culture***

A375 (human amelanotic melanoma) cells were purchased from American Type Culture Collection (Manassas, VA, USA) and cultured in RPMI-1640 medium (Sigma-Aldrich, St. Louis, MO, USA) supplemented with 10% foetal calf serum (Invitrogen, CA, USA) and 2 mM l-glutamine (Sigma). Cells were maintained in 175 cm^2^ flasks in at 37°C in 5% CO_2_:95% atmosphere humidified incubator and grown to sub-confluent monolayers before being detached using trypsin for use in animal models.

#### ***Cell uptake and inhibition studies***

In 24-well culture plates, 2.5 × 10^5^ cells were seeded in complete medium and left to attach overnight. The following day, the number of cells per well was counted in triplicate. Before incubation with the radiotracer, the growth media was removed and the cells were washed once with warm PBS to remove all traces of growth media. ^18^F-SIG343 or ^18^F-SIG353 was formulated in warm PBS containing 0.1% Tween-80. Freshly prepared radiotracer (0.37 MBq in 500 μL) was added and the cells were incubated at 37°C for 2, 15, 30, 60 and 120 min. Uptake was terminated by removing the tracer solution and washing cells with ice-cold PBS. Subsequently, the cells were lysed with 500 μL of 0.2 N NaOH. The radioactivity in the cell lysate was measured with a Wallac 1480 γ-counter (PerkinElmer, MA, USA). The results were expressed as percentage of applied dose per 1 × 10^5^ cells. All activities were corrected for decay. Optimal uptake time will be selected for inhibition cell uptake studies.

The specific uptake of the radiotracers into the cancer cells was examined in the presence of σ ligands as competitors (final concentration 5 μM): (+)-pentazocine (σ_1_), haloperidol (non-selective σ_1_/σ_2_) and unlabelled SIG343 or SIG353 (σ_2_). Cells were prepared as described for uptake studies. Prior to the addition of the radiotracer, 400 μL of blocking drug (6.25 μmol/L in PBS) or PBS (for controls) was added to the wells. Freshly prepared radiotracer (0.37 MBq in 100 μL) was added, and the cells were incubated at 37°C for 15 min as the optimal time previously determined by the cell uptake studies. Uptake was terminated as described for uptake studies. The percentage of uptake in the treatment groups relative to the control group was determined.

### Ethics

Animal experiments were performed according to the Australian Code of Practice for the Care and Use of Animals for Scientific Purposes and were approved by the ANSTO Animal Care and Ethics Committee.

### *In vivo* studies

#### ***A375 tumour-bearing mice model***

Female Balb/C nude mice aged 5 to 6 weeks old were obtained from the Animal Resource Centre (Perth, Australia). The animals were kept at a constant temperature of 22°C ± 2°C on a 12/12 h light/dark cycle with lights on at 09:00 am. Food and water were freely available. After a week of acclimatisation, mice were injected subcutaneously in the left flank with 1 × 10^6^ A375 human amelanotic melanoma cells, in 100 μL of Ca^2+^/Mg^2+^-free phosphate buffered saline. The procedure was performed without anaesthetic. For *in vivo* studies, A375 tumour-bearing mice were used 26 days after tumour inoculation.

#### ***Biodistribution studies***

Tumour-bearing mice were used to examine the tissue distribution of radioactivity after intravenous injection into the tail vein of 1 MBq of ^18^F-SIG343 or ^18^F-SIG353 in 100 μL of saline. At 15, 30, 60, and 120 min post injection of the radiotracer, groups of mice (*n* = 5) were sacrificed by CO_2_ administration followed by cervical fracture and dissection. Selected organs were weighed, and the radioactivity was measured using a γ-counter. The percentage of injected dose (%ID) was calculated by comparison with suitable dilutions of the injected dose. Radioactivity concentrations were expressed as percentage of injected dose per gram of wet tissue (%ID/g), assuming a uniform density of 1 g/cm^3^. Data were corrected for the radioactivity decay and tail injected dose and normalised for the standard mouse body weight (20 g). The remaining activity in the carcass was also determined in order to obtain the total activity in the mouse (background activity) at each time point.

#### ***Blocking studies***

Blocking studies were performed to investigate the specific uptake of the tracer via σ_2_ receptor mechanism. Haloperidol and the unlabelled compound SIG343 or SIG353 (1 mg/kg) were administered by intravenous injection 5 min prior to radiotracer administration. Control mice received saline only. Five minutes after injection of the blocking drug, ^18^F-SIG343 or ^18^F-SIG353 (1 MBq/100 μL of saline) was injected, and groups of mice (*n* = 5) were sacrificed 30 min post injection of the radiotracer. Organs were processed as described for the biodistribution study. Radioactivity concentrations in the organs in the treatment groups were compared to that measured in control mice.

#### ***Metabolite studies***

The amount of intact ^18^F-SIG343 and ^18^F-SIG353 in the plasma, urine, tumour and brain cortex was quantified by thin layer chromatography (TLC) and radio-HPLC analysis. Mice were injected with 20 MBq of the radiotracer in 100 μL of saline. Mice were sacrificed 15, 60 and 120 min post injection of radiotracer. Whole organ samples of brain cortex and tumour samples (10 to 60 mg, minced) were added to unlabelled SIG343 or SIG353 (5 μL; 1 mg/mL), KF (5 μL; 1 mg/mL), MeCN (0.3 mL) and water (0.2 mL). Samples were exposed to an ultrasonic probe (Ultrasonic processor, Misonix Inc., Farmingdale, NY, USA) for 2 min before being centrifuged (5,000 rpm, 5 min). Plasma (50 μL) or urine (10 μL) samples were added to unlabelled SIG343 or SIG353, KF and 0.5 mL MeCN. Plasma samples were centrifuged (Heraeus Biofuge PrimoR, Thermo Fisher Scientific, Hudson, NH, USA; 5,000 rpm, 5 min), and the supernatant was removed and the radioactivity of the precipitated pellets was measured using a gamma counter to determine the extraction efficiency. If necessary, multiple extractions were performed to ensure maximum recovery of the radioactivity. An appropriate amount of the supernatant, based on the activity level (cpm), was collected (approximately 100 μL), diluted with water (up to 1.5 mL) for HPLC analysis or evaporated to dryness under vacuum for TLC analysis.

The TLC sample was reconstituted in methanol (25 to 50 μL) and mixed before being applied to the concentrating zone of the silica TLC plate. In a separate lane, the corresponding ^18^F-labelled and ^19^F-standard was also spotted. The TLC solvent systems of EtOAc/MeOH (70:30) were utilised for ^18^F-SIG343 (rf 0.50) and ^18^F-SIG353 (rf 0.55). The UV of the standard was identified using a UV lamp, while the movement and integration of the radioactive spots were visualised and measured using a phosphorimager (BAS 2500 Phosphorimager, Fujifilm, Tokyo, Japan) with Fujifilm Multigage 3.0 software. The intact radiotracer was identified as the radioactive spot containing the identical rf value to the corresponding ^19^F-standard seen under the UV lamp. The integration of the active spot in relation to all the activity in the TLC lane gave the percentage of intact radiotracer.

Radio-HPLC analysis was performed following the method of Hilton et al. [52]. A pre-column (Waters Oasis HLB, 25 μm, 20 × 3.9 mm) and a reversed phase HPLC column (Phenomenex Bondclone C18, 10 μm, 250 × 4.6 mm or Phenomenex Synergi Max-RP 80A C18, 4 μm, 250 × 4.6 mm) in series, with a switching valve between columns was utilised. The pre-column was washed with 1% acetonitrile in water for 3 min at 1.5 mL/min and then the solvent direction was switched to include the HPLC column. Both columns in series were then eluted over 10 min. The radioactivity peak corresponding to the authentic radiotracer was compared to the total activity registered in the radiochromatogram to give the fraction of unchanged radiotracer in the sample.

#### ***PET/CT imaging studies***

The mouse was anaesthetised via inhalant isoflurane (Forthane, Abbott Laboratories, IL, USA) (5% induction, 1% to 3% maintenance in 200 mL/min oxygen). Respiration and heart rates of the animal were monitored (BioVet; m2m Imaging Corp, Cleveland, OH, USA) during the entire scanning period. The core body temperature of the animals was maintained via a temperature-controlled heating pad. The mouse was injected intravenously with 4.6 to 16.7 MBq of ^18^F-SIG343 or ^18^F-SIG353, with a constant mass of the unlabelled compounds of 0.06 nmol in 100 μL of saline (*n* = 3). A 120-min PET scan was performed on an Inveon multimodality positron emission tomography-computed tomography (PET/CT) system (Siemens Medical Solutions, Knoxville, TN, USA) followed by a 10-min CT scan on each subject for anatomical information. Image acquisition commenced simultaneously with radiotracer injection. The data was histogrammed into 25 consecutive frames, and activity volumes were reconstructed with an iterative reconstruction (OSEM/MAP) including attenuation and scatter correction, achieving a reconstructed spatial resolution of 1.5 mm [53]. Briefly, each individual PET scan was co-registered to its respective CT (automatic and visual quality control) (Anatomist/Brainvisa, version 3.1.4). Regional activity data (Bq/cc) were extracted from nine selected volumes/regions of interest (VOIs/ROIs) where σ receptor distribution was reported and the tail and expressed as percentage of injected dose per cubic centimetres (%ID/cc).

### Data and statistical analyses

Significant outliers were identified by Grubb tests and removed from the raw data set. Subsequently, data were analysed using the GraphPad Prism 5.04 (GraphPad Software Inc., San Diego, CA, USA) statistical package software. Significance was set at *P* ≤ 0.05 for all statistical analyses.

In the cell uptake blocking studies, separate one-way ANOVAs [drug treatment as a factor (control, (+)-pentazocine, haloperidol and unlabelled SIG343 or SIG353)], followed by Bonferroni's *post hoc* tests were performed to statistically determine the significant changes in the uptake percentage of the radiotracers into the cells compared to controls.

Selected tumour-to-organ (tumour-to-blood and tumour-to-muscle) uptake ratios (TORs), were calculated by dividing the tumour radioactivity concentration (%ID/g) by the radioactivity concentration in the respective organ at time *t*. Comparisons of organ radioactivity concentrations (%ID/g) and TORs in the biodistribution studies or %ID/cc in PET imaging studies between the two radiotracers were performed using separate unpaired, two-tailed Student's *t* tests, matched organs and time points.

Meanwhile, for the blocking studies, separate within-group two-way ANOVAs [drug treatment (control, haloperidol and unlabelled SIG343 or SIG353) × regions] followed by Bonferroni's *post hoc* tests were performed for each radiotracer to determine any statistically significant changes in the uptake (%ID/g) amongst selected ROIs compared to the control.

## Results

### Chemistry

**Scheme 1:**
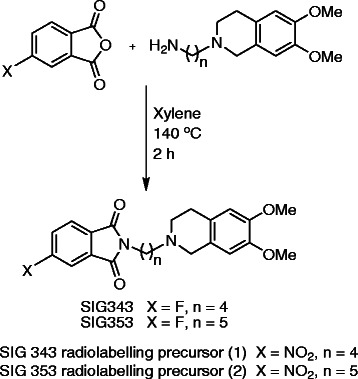
Synthesis of sigma phthalimide derivatives SIG343, SIG353 and their radiolabelling precursors.

The synthesis of SIG343 and SIG353 and their respective nitro-radiolabelling precursor was via a condensation of the appropriate phthalic anhydride with either 4-(6,7-dimethoxy-3,4-dihydroisoquinolin-2(1H)-yl)butan-1-amine for the SIG343 pair or 5-(6,7-dimethoxy-3,4-dihydroisoquinolin-2(1H)-yl)pentan-1-amine for the SIG353 pair (Scheme 1). Recrystallisation of these compounds from aqueous ethanol gave these compounds as crystals in greater than 80% yield. Interestingly, SIG343 and SIG353 were stable colourless crystals, whereas the nitro equivalents of these compounds were both initially bright orange crystals that darkened to brown crystals over a week on the bench. Stability, as indicated by the bright orange state of the nitro precursors, was maintained over a year by storing them under nitrogen in a dark vial in the freezer.

### Radiochemistry

**Scheme 2:**
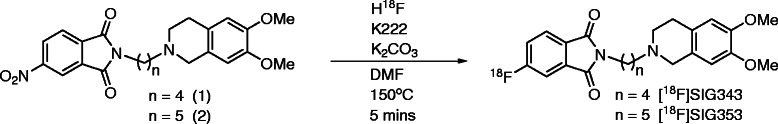
**Radiosynthesis of **^**18**^**F-SIG343 and **^**18**^**F-SIG353.**

Radiotracers ^18^F-SIG343 and ^18^F-SIG353 were synthesised by aromatic nucleophilic substitution of their respective nitro precursors, (*1*) and (*2*) (Scheme 2), using potassium carbonate and Kryptofix 222 in DMF at 140°C for 5 min. The isolated collected radiolabelled yields were similar for each radiotracer, 18 ± 5% (*n* = 13) and 18 ± 7% (*n* = 13) (non-decay corrected) for ^18^F-SIG343 and ^18^F-SIG353, respectively. Controlling the basicity of the reaction was an important parameter that affected the yield. Levels of potassium carbonate above 1 mg generally resulted in lower yields possibly by inducing hydroxide formation from trace amounts of water in the reaction, with subsequent ring opening of the phthalimido ring or acting as a competitor of the fluoride.

The radiochemical purity of both radiotracers was greater than 97% over 3 h in a saline solution, while specific activity was greater than 82 GBq/μmol at time of injection for all studies.

### *In vitro* studies

#### ***Radioreceptor binding assays***

SIG343 and SIG353 were evaluated using competitive inhibition experiments to determine their binding affinity (*K*_i_) to the σ_1_ and σ_2_ receptors and a number of other neuroreceptors for their selectivity. The radioreceptor binding studies showed high affinity and high selectivity for σ_2_ receptors, with 200- and 110-fold selectivity for σ_2_ receptors compared to σ_1_, for SIG343 and SIG353, respectively. The compounds did not show any cross reactivity for the other neuroreceptors examined (*IC*_50_ > 10,000 nM). The *K*_i_ or *IC*_50_ values and log*P* values are shown in Table 2.

**Table 2:** ***In vitro *** **characteristics of SIG343 and SIG353**

**Parameters**	**Compound**	
	**SIG343**	**SIG353**
Affinity (nM)		
σ_1_^a^	1,600	266
σ_2_^a^	8	2.4
Opioid, non-selective^b^	>10^4^	>10^4^
Dopamine, non-selective^b^	>10^4^	>10^4^
Serotonin, non-selective^b^	>10^4^	>10^4^
CB_1_, non-selective^b^	>10^4^	>10^4^
Muscarinic, non-selective^b^	>10^4^	>10^4^
Selectivity		
σ_2_:σ_1_	200	110
Lipophilicity		
Log*P*^b^	3.11 ± 0.08	3.47 ± 0.09

^a^*K*_i_, determined by ANSTO LifeSciences; ^b^*IC*_50_, determined by Caliper LifeSciences.

#### ***Cell uptake and inhibition studies***

**Figure 1:**
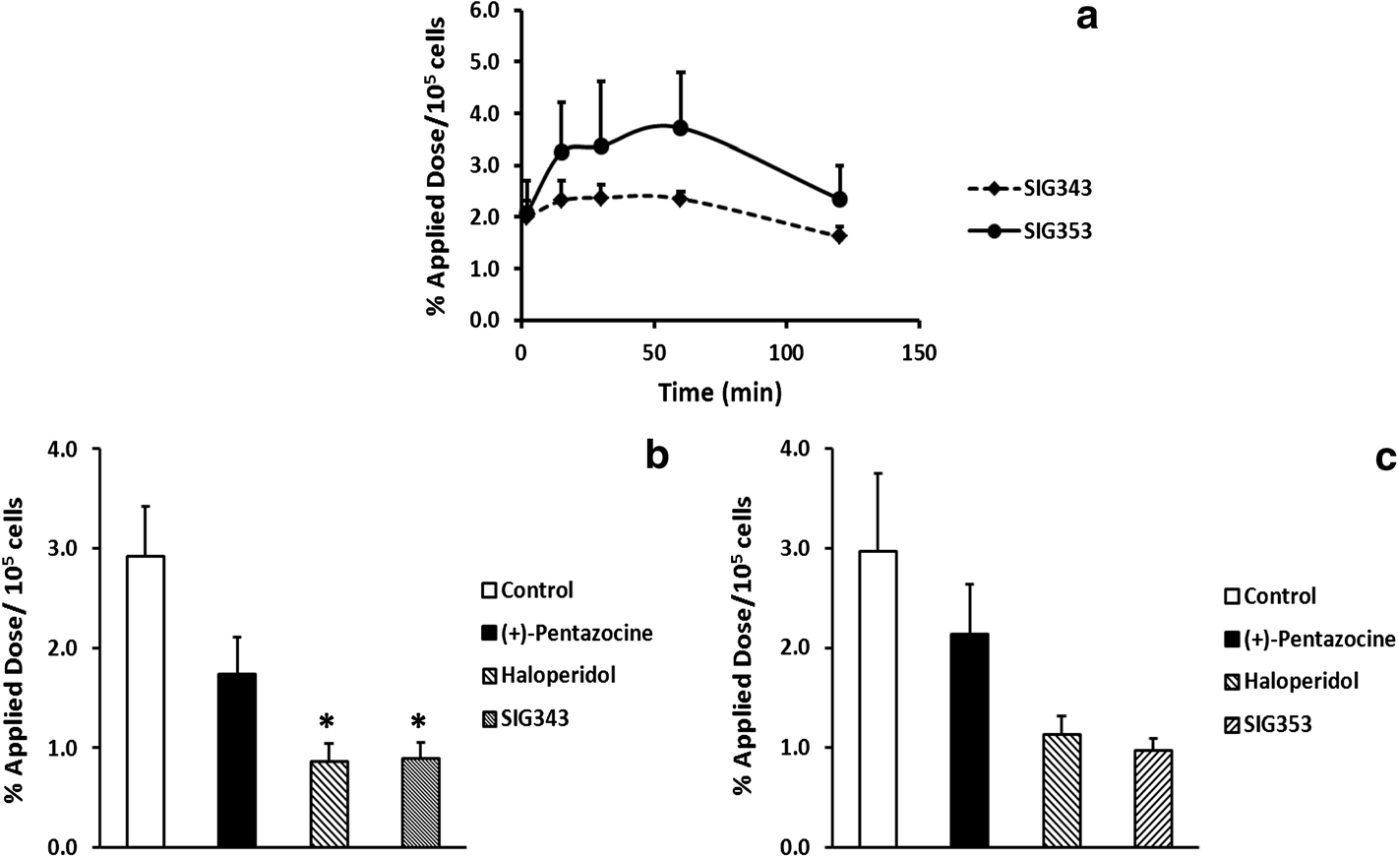
**Cell uptake and inhibition studies. (a)** Cell uptake studies over 120 min of ^18^F-SIG343 and ^18^F-SIG343. Cell uptake inhibition studies of ^18^F-SIG343 **(b)** and ^18^F-SIG353 **(c)** in the presence of competing σ ligands: (+)-pentazocine, haloperidol, unlabelled SIG343 or SIG353 (5 μM). Results were expressed as percentage of applied dose per 1 × 10^5^ of A375 amelanotic human cells with decay correction (means ± SD, *n* = 3). *P <0.05 drug treatment versus control (one-way ANOVAs with treatment as a factor, followed by Bonferroni's *post hoc* tests).

The uptake of ^18^F-SIG343 and ^18^F-SIG353 in the A375 amelanotic human cell lines was explored over 120 min. Uptake in A375 cells for both radiotracers was found to be rapid (approximately 2%, after 2 min), peaked at 3% to 3.5% between 15 and 60 min for ^18^F-SIG353 and plateaued at 2% for ^18^F-SIG343 for the same period. Uptake slightly declined after 60 min to 1.6% to 2.3% for ^18^F-SIG343 and ^18^F-SIG353, respectively (Figure 1a). In parallel studies, the specific cell uptake due to σ receptor binding was verified by the presence of a variety of σ ligands including (+)-pentazocine (σ_1_), haloperidol (non-selective σ_1_/σ_2_) and unlabelled SIG343 and SIG353 (σ_2_). One-way analysis of variance (ANOVA) analysis (treatment as a factor) indicated an overall significant variation in the cell uptake values amongst all treatment groups for ^18^F-SIG343 (*F*_3,8_ = 2.78, *P* < 0.01). Bonferroni’s *post hoc* tests revealed the cell uptake of ^18^F-SIG343 was significantly reduced by haloperidol (-72%, *P* < 0.05) and unlabelled SIG343 (-69%, *P* < 0.05), whereas (+)-pentazocine produced no significant blocking effect on ^18^F-SIG343 cell uptake compared to the controls (Figure 1b). In contrast, the uptake of ^18^F-SIG353 into A375 cells was not significantly reduced by any of the competing σ ligands (*F*_3,8_ = 2.59, *P* = 0.0579) (Figure 1c) using the same statistical assessments.

### *In vivo* studies

#### ***Biodistribution studies***

**Figure 2:**
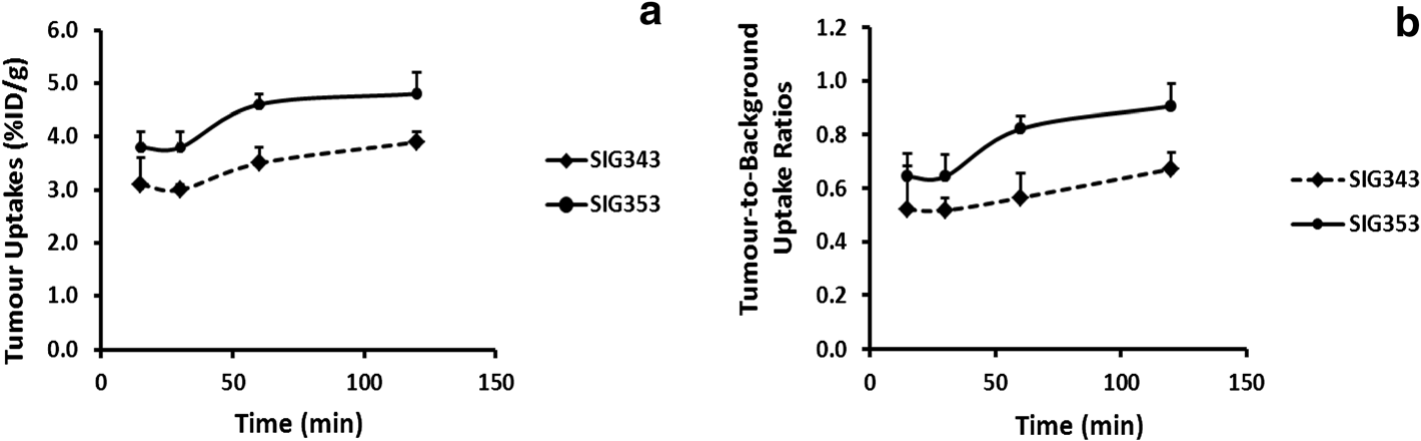
**Comparisons of the uptakes in tumour (a) and tumour-to-background uptake ratios (b) of **^**18**^**F-SIG343 and **^**18**^**F-SIG353.** Results were presented as %ID/g in the tumour and the uptake in the tumour compared to the residual activity remained in the body (tumour-to-background uptake ratios) at 15, 30, 60 and 120 min. Data are derived from the biodistribution studies in A373 tumour-bearing mice (means ± SEM, *n* = 5). *P < 0.05, **P < 0.01, ***P < 0.001 treatment versus control (unpaired, two-tailed Student's *t* test).

The tissue distribution of radioactivity in Balb/C nude mice bearing A375 tumours following intravenous administration of ^18^F-SIG343 and ^18^F-SIG353 is summarised in Table 3. In general, both radiotracers exhibited similar biodistribution profiles. The highest initial uptake was observed in the liver, kidneys, lungs and pancreas for both radiotracers. In organs that are known to express the σ_2_ receptors [13,15,54,55], the clearance of the radiotracer was only observed in the brain, lungs, kidneys, spleen and heart. Moderately high uptake values in the tumour (4% to 5%) were observed for both radiotracers (Figure 2a,b). Radioactivity accumulated in the tumour and ovaries over time, while uptake in the liver and pancreas continued to increase for the duration of the study resulting in the lack of clearance of the radiotracers. Uptake in the bone was measurable and remained stable over time, suggesting no significant *in vivo* de-fluorination and subsequent fluoride bone uptake.

**Table 3:** **Biodistribution of **^**18**^**F-SIG343 and **^**18**^**F-SIG353 in representative tissues (decay corrected)**

**Compound**	**Time (min)**	**Brain**	**Liver**	**Spleen**	**Kidneys**	**Femur**	**Lungs**	**Heart**	**Blood**	**Pancreas**	**Ovaries**	**Tumour**
^18^F-SIG343	15	9.6 ± 0.2^***^	16.0 ± 0.9^**^	14.6 ± 1.2	26.1 ± 0.6	4.5 ± 0.4	30.3 ± 1.2^***^	5.5 ± 0.4	0.9 ± 0.1	42.9 ± 2.8^*^	10.8 ± 1.8	3.1 ± 0.5
	30	8.8 ± 0.6^***^	21.9 ± 0.8^***^	15.2 ± 1.1^*^	19.8 ± 0.9^***^	4.3 ± 0.2	17.4 ± 0.7^***^	3.5 ± 0.2^**^	0.7 ± 0.04	50.2 ± 2.0^***^	14.0 ± 2.2	3.0 ± 0.1^*^
	60	7.1 ± 0.4^***^	15.2 ± 1.1^***^	12.8 ± 0.9^***^	12.0 ± 0.6^**^	3.8 ± 0.1	9.9 ± 0.3^***^	1.9 ± 0.04^**^	0.6 ± 0.1	64.4 ± 3.5^***^	13.5 ± 2.2	3.5 ± 0.3^*^
	120	5.2 ± 0.7^***^	19.8 ± 0.9^*^	8.5 ± 0.2^***^	7.5 ± 0.4	3.2 ± 0.1	5.0 ± 0.3	1.1 ± 0.1	0.4 ± 0.01	61.7 ± 3.4^***^	11.5 ± 1.4	3.9 ± 0.2
^18^F-SIG353	15	4.4 ± 0.2	23.5 ± 2.0	15.7 ± 1.1	23.4 ± 2.0	5.3 ± 0.3	17.4 ± 0.5	4.5 ± 0.2	1.2 ± 0.03	56.4 ± 4.7	10.0 ± 0.9	3.8 ± 0.3
	30	2.3 ± 0.1	33.0 ± 1.8	12.0 ± 0.6	13.3 ± 0.8	4.7 ± 0.2	8.6 ± 0.4	2.5 ± 0.1	1.0 ± 0.1	72.8 ± 2.4	11.8 ± 1.5	3.8 ± 0.3
	60	1.3 ± 0.01	32.6 ± 1.0	7.7 ± 0.4	8.7 ± 0.3	3.8 ± 0.2	6.1 ± 0.1	1.7 ± 0.02	0.7 ± 0.02	94.3 ± 4.2	9.6 ± 1.5	4.6 ± 0.2
	120	0.8 ± 0.02	24.1 ± 1.4	5.5 ± 0.2	7.1 ± 0.2	3.9 ± 0.1	4.7 ± 0.2	1.3 ± 0.1	0.7 ± 0.03	119.2 ± 1.6	12.2 ± 3.1	4.8 ± 0.4

Data are presented as the means of percentage of injected dose per g of tissue (%ID/g ± SEM, *n* = 5). Comparisons of ^18^F-SIG343 vs. ^18^F-SIG353, unpaired two-tailed Student's t test, matched organ and time point. SEM scanning electron microscopy *0.01 < *P* < 0.05 (significant); **0.001 < *P* < 0.01 (very significant); ***P < 0.001 (extremely significant).

Despite the similarities, the two radioligands exhibited some differences in the extent and the rate of uptake. For instance, a significantly higher uptake of ^18^F-SIG343 compared to that of ^18^F-SIG353 was observed in the brain, spleen, heart, kidney, lung, and tumour between 30 and 60 min (0.0001 < *P*s < 0.05). In contrast, the uptake of ^18^F-SIG353 was significantly higher than ^18^F-SIG343 in the liver and pancreas for the same period. At 120 min, the uptake in the lungs, kidneys, tumour and heart was not different between the two radiotracers. The uptake in the ovaries was not different between the two radiotracers at any time point. In the brain, clearance was found to be more rapid for ^18^F-SIG353, whereas the lung clearance was faster for ^18^F-SIG343.

**Figure 3:**
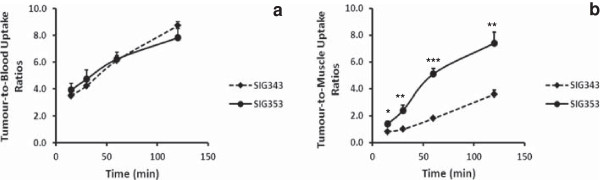
**Comparisons of the tumour-to-blood (a) and tumour-to-muscle (b) uptake ratios of **^**18**^**F-SIG343 and **^**18**^**F-SIG353.** Results were presented as the ratios of the radioconcentration (%ID/g) in the tumour and in the blood or muscle at 15, 30, 60 and 120 min. Data are derived from the biodistribution studies in A373 tumour-bearing mice (means ± SEM, *n* = 5). *P < 0.05, **P < 0.01, ***P < 0.001 treatment versus control (unpaired, two-tailed Student's *t* test).

Tumour-to-blood and tumour-to-muscle uptake ratios were derived from the biodistribution data. Both ^18^F-SIG343 and ^18^F-SIG353 showed similar tumour-to-blood uptake ratios (eight- to ninefold at 120 min), whereas ^18^F-SIG353 had significantly higher tumour-to-muscle uptake ratios compared to that of ^18^F-SIG343 at any time point (0.0001 < *P*s <0.05) (Figure 3a). The highest tumour-to-muscle uptake ratio was observed at 120 min for ^18^F-SIG353 (sevenfold) (Figure 3b).

#### ***Blocking studies***

The degree of *in vivo* specific binding of ^18^F-SIG343 and ^18^F-SIG353 due to σ_2_ receptors was examined using blocking studies performed in the same animal model. The blocking effect on radiotracer uptake following pre-administration of haloperidol (non-selective σ_2_) and unlabelled SIG343 or SIG353 (σ_2_) as competitors was observed in various tissues at 30-min post injection of the radiotracer.

**Figure 4:**
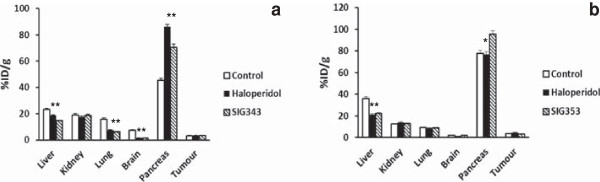
**Blocking studies of **^**18**^**F-SIG343 and **^**18**^**F-SIG353.** Blocking studies of ^18^F-SIG343 **(a)** and ^18^F-SIG353 **(b)** in the presence of competing σ_2_ ligands: haloperidol, unlabelled SIG343 or SIG353 (1 mg/kg**)**, respectively. Results were expressed as radioconcentration of the tracers in the organs in the treatment group compared to controls (means ± SEM, *n* = 5). *P <0.001 drug treatment versus control (within-group two-way ANOVAs (treatment × regions), followed by Bonferroni's *post hoc* tests).

Nine organs and tissues that are known to have σ receptors that are widely distributed including the brain, liver, spleen, kidneys, lungs, heart, pancreas, ovaries and tumour were included in statistical analyses. Separate within two-way ANOVAs (drug treatment × regions) examining the blocking effect of pre-administration of competing σ ligands on ^18^F-SIG343 organ distribution. Significant main effects of treatment (*F*_2,107_ = 11.38, *P* < 0.0001), regions (*F*_8,107_ = 1,249, *P* < 0.0001) and the significant interaction between the two factors (*F*_16,107_ = 57.38, *P* < 0.0001) were also noted. Similar significant blocking was found in almost organs expressing σ receptor in both haloperidol- and unlabelled SIG343-treated groups including brain (↓78% to 79%, *P*s < 0.001), liver (↓20% to 37%, *P*s < 0.001), spleen (↓45% to 52%, *P*s < 0.001), and lungs (↓53% to 60%, *P*s < 0.001), respectively (Figure 4a).

On the other hand, in similarly designed analysis, separate 2-way ANOVAs were also carried out to evaluate the blocking effect of competing σ ligands on ^18^F-SIG353 regional distribution. Significant main effects of treatment (*F*_2,107_ = 7.38, *P* < 0.001), regions (*F*_8,107_ = 691.5, *P* < 0.0001) and significant interaction between the two variables (*F*_16,107_ = 11.8, *P* < 0.0001) were observed. Bonferroni *post hoc* revealed only significant blocking occurred in the liver (↓38% to 41%, *P*s < 0.001) for both haloperidol and unlabelled SIG353 treatment groups, respectively. Uptake of ^18^F-SIG353, however, increased in the pancreas for the unlabelled SIG353-treated group (↑37%, *P* < 0.001) (Figure 4b).

#### ***Metabolite studies***

The stability of the ^18^F-SIG343 and ^18^F-SIG353 *in vivo* was studied over 120 min, after i.v. injection in tumour, brain cortex, plasma and urine samples. The results shown in Table 4 suggest that both radiotracers have similar stabilities *in vivo*. The determination of the unchanged radiotracer was carried out by radio-HPLC and radio-TLC. Both analytical methods generated results in concordance with each other presenting a similar trend of *in vivo* stability.

**Table 4:** **Percentage of the unchanged form in organs and associated sample recovery of **^**18**^**F-SIG343 and **^**18**^**F-SIG353**

**Compound**	**Time (min)**	**Unchanged form (%)**^**a **^**[sample recovery (%)]**^**b**^			
		**Tumour**	**Cortex**	**Plasma**	**Urine**
^18^F-SIG343	15	79 ± 4.6 (86)	87 ± 3.2 (92)	49 ± 7.5 (89 ± 14.4)	13 ± 1.2
	60	85 ± 3.1 (90)	83 ± 0.8 (88)	23 ± 3.8 (86 ± 16.4)	15 ± 4.3
	120	80 ± 5.0 (86)	86 ± 1.0 (90)	16 ± 2.8 (91 ± 9.0)	11 ± 5.4
^18^F-SIG353	15	81 ± 4.5 (86)	85 (91)	60 ± 3.0 (92 ± 6.8)	5 ± 1.3
	60	85 ± 2.4 (90)	87 (93)	27 ± 5.1 (88 ± 10.7)	4 ± 0.4
	120	65 ± 8.9 (81)	IR	17 ± 2.9 (94 ± 5.3)	5 ± 1.2

^a^Means ± SD (*n* =3); ^b^the recovery of the radioactivity. *IR* insufficient radioactivity for analysis.

Following ultrasonic disruption and centrifugation of tissue, at least 81% of radioactivity from the tumour and brain cortex was recovered in the supernatant from samples collected at 15, 60 and 120 min. However, ^18^F-SIG353 radioactivity collected from brain at 15 and 60 min was too low for detection radio-HPLC so only the radio-TLC data are included for this tissue. Radioactivity collected from brain at 120 min was too low to detect by radio-HPLC and radio-TLC, therefore this sample was not analysed. More than 80% of recovered radioactivity was intact ^18^F-SIG343 in the tumour and brain tissues after 120 min. On the other hand, ^18^F-SIG353 was not as stable in the tumour with intact radiotracer reduced to 65% at 120 min. However, there was no significant decrease in the percentage of unchanged radiotracers found (less than 10%) in the above-mentioned organs, at 120 min post injection.

In plasma, rapid decrease of unchanged radiotracers was observed; the percentage of unchanged ^18^F-SIG343 and ^18^F-SIG353 was reduced from 60% at 15 min to 17% at 120 min. In urine, unchanged ^18^F-SIG343 was rapidly reduced to 13% at 15 min while only 5% of radioactivity was intact ^18^F-SIG353 at 15 min.

#### ***PET studies***

**Figure 5:**
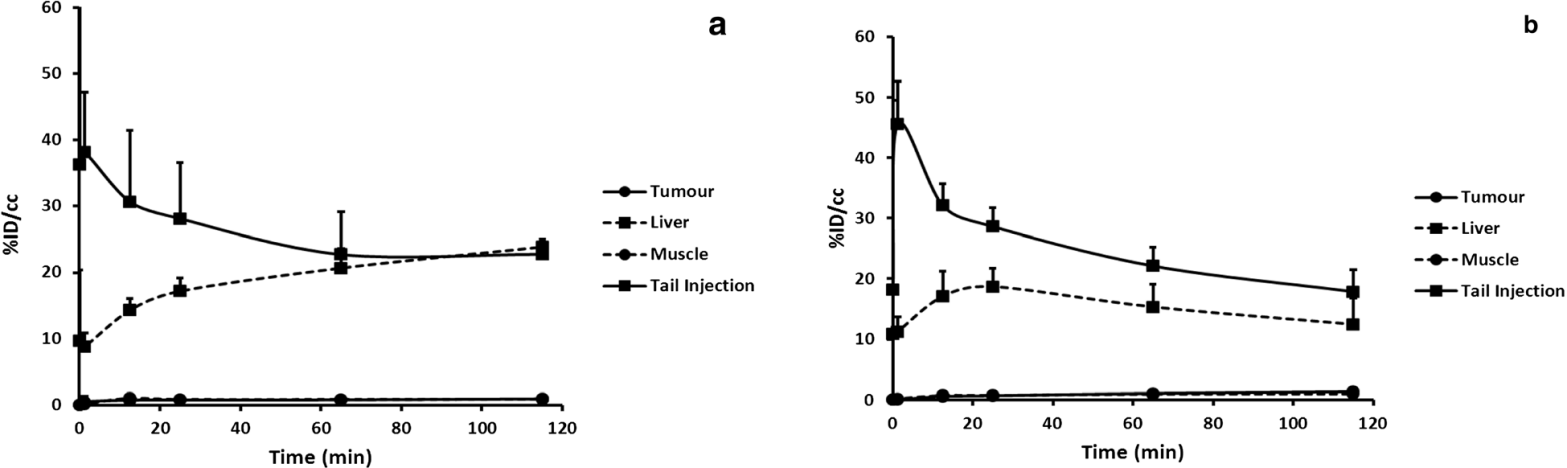
**Time-activity curves of **^**18**^**F-SIG343 (a) and **^**18**^**F-SIG353 (b) in selected organs.** Results were expressed as Activity (%ID/cc) over Time (min) (means ± SD, *n* = 3). Mice were injected intravenously with 4.6-16.7 MBq of ^18^F-SIG343 or ^18^F-SIG353, with a constant tracer mass of the unlabelled compound of 0.06 nmol/100 μL of saline. A 120-min PET scan was performed on an Inveon PET/CT system, followed by a 10-min CT scan on each subject for anatomical information.

Results from the PET imaging studies showed high uptake in the chest cavity for both radiotracers, with continuous accumulation in the liver. Low uptakes were observed in the tumour, with ^18^F-SIG353 having higher uptake compared to ^18^F-SIG343, (*P* < 0.05) resulting low tumour-to-background contrast. Time-activity curves of ^18^F-SIG343 and ^18^F-SIG353 of selected organs are presented in Figure 5a,b.

## Discussion

In the current study, radioreceptor binding assays identified two lead σ_2_ ligands, SIG343 and SIG353, from a series of phthalimide derivatives that have high affinity and are highly selective for σ_2_ receptors. From literature, it is interesting to note that the structure designed for the σ receptor also shows affinity for the related dopamine receptors [56,57]. However, small structural differences can greatly influence a compounds affinity and selectivity for any of the receptors. As a result, our *in vitro* data indicated that the compounds have no affinity for dopamine receptors.

The potential of these tracers as imaging agents for identification of σ_2_ receptor localisation *in vivo*, particularly in tumours, was explored. A number of cancer cell lines that express various concentrations of σ receptors have been described; however, the level of expression of the two σ subtypes was often not reported [39]. Amongst these cell lines, MDA-MB-231 and PC-3 cell lines were reported to express 16% and 33% of σ_2_ receptors, respectively [27]. The A375 human amelanotic cell line was reported to express high concentration of σ_2_ receptors, approximately 100 times higher than that of the σ_1_ subtype [27]. The relationship of σ_2_ receptors and cellular proliferation was explored and demonstrated that proliferative cells have ten times more σ_2_ receptors than quiescent cells, although full down-regulation of the σ_2_ receptor was reached only several (>3) days after the cells had become quiescent [32]. Thus, the half-life of the σ_2_ receptor appeared to be rather long (>12 h) [39]. In our study we seeded the cell cultures overnight after the cells had reached 80% confluence (i.e. proliferative phase) to ensure the cells were expressing the maximum amount of σ_2_ receptors in the cells, also to optimise signal-to-noise ratio in cell uptake studies. The results from the cell uptake inhibition studies are in agreement with the *in vitro* receptor binding data confirming that SIG343 has a higher selectivity for σ_2_ receptors, compared to SIG353.

Biodistribution studies in tumoured mice indicated that statistically significant uptake of ^18^F-SIG343 and ^18^F-SIG353 were retained in the organs known to express high densities of σ receptors such as the brain, liver, kidneys, lungs, pancreas and ovaries. As the uptake of the radiotracers in the liver and pancreas was still increasing, it is possible that the terminal half-lives of these ligands are longer than 120 min which is beyond the last time point of the study. Lack of clearance of the tracers observed at 120 min may be attributable to either continuing accumulation of the radiotracer and/or its radioactive metabolite in the liver and pancreas.

Despite some similarities in distribution profiles and clearance in the organs, the two radiotracers exhibited some differences in the extent and the rate of uptake. For instance, a significantly higher uptake of ^18^F-SIG343 compared to that of ^18^F-SIG353 was observed in the brain and the lung. However, the brain clearance was more rapid for ^18^F-SIG353, and the lung clearance was faster for ^18^F-SIG343. High to moderately high brain uptake of the radiotracers together with their appropriate lipophilicity indicated that the radiotracers are also suitable as imaging agent for receptors in the brain.

Moderately high and prolonged tumour uptake, 3% to 4% and 4% to 5% for ^18^F-SIG343 and ^18^F-SIG353, respectively, throughout the study and up to 120 min, showed promising tumour imaging potential for these radiotracers. Both radiotracers showed similar trends of increasing tumour-to-blood and tumour-to-muscle uptake ratios over time. Selective σ_2_^18^F-radioligands for PET imaging from the literature were reported to have tumour-to-muscle ratios ranging from 2 to 8 at 120 min in various tumoured mouse models (Table 1). Tumour-to-muscle uptake ratios of ^18^F-SIG343 and ^18^F-SIG353 are 4 and 7 at 120 min respectively, which are comparable to those reported. Similarly, the tumour-to-blood uptake ratios were high at 8 to 9 for both radiotracers as a result of the increasing radiotracer uptake in tumour over time, and the radioactivity clearance from the systemic circulation (increasing tumour-to-blood concentration ratios) or from the muscle (increasing tumour-to-muscle uptake ratios) over time. Bone uptake of both radiotracers was low and has steadily declined over time which indicates no significant *in vivo* de-fluorination and subsequent fluoride bone uptake.

In the blocking study, we used only selective σ_2_ ligands as competitors as the σ_2_ specificity of these radiotracers had been previously established in naïve animals (manuscript in preparation). The competing drugs (1 mg/kg in 100 μL) including haloperidol (Ki(σ_2_) = 20 nM) and unlabelled SIG343 and SIG353 (Ki(σ_2_) = 8 and 2.7 nM, respectively) were administered 5 min prior to radiotracer injection. The concentration used deems to be in excess for all compounds used (i.e. 532, 485 and 469 nM for haloperidol, SIG343 and SIG353, respectively) which are at least 50 times greater than the *K*_i_ values of the radiotracers for σ_2_ receptors to ensure a complete saturation of receptor occupancy.

It is interesting to note that the uptake in the pancreas were found to increase (↑55% to 89%, *P*s < 0.001) in both treatment groups (haloperidol-treated and unlabelled SIG343-treated) compared to controls for ^18^F-SIG343. However, this increased uptake phenomenon in the pancreas was only observed in the unlabelled SIG353-treated (↑37%, *P* < 0.001), but not in the haloperidol-treated group, for ^18^F-SIG353 (*P* > 0.05). It is possible that pancreas is a site of radioactive metabolite accumulation, and the metabolism is a dynamic process which would make it difficult to measure specific binding in this organ. This observation was also evident in, and supported by, the biodistribution data which showed that the uptake in the liver and pancreas was constantly increasing over time. Although there was no significant increase in radioactivity of the tracer in the blood or bone as a result of the blocking drug treatment (data not shown), based on the increased radiotracer uptake values over time in many organs expressing σ receptors, we hypothesise that the continuing increase in pancreas and liver uptake is likely due to an increase in radioactive metabolites, rather than specific σ uptake.

Amongst the competing σ_2_ ligands, the pharmacokinetics of unlabelled SIG343 and SIG353 is unknown and limited in rodent studies, particularly in mice for haloperidol, and the only available pharmacokinetic data of haloperidol are those in rats [58] (terminal half-life of 1.5 h, intravenous bolus). Since haloperidol was reported to have linear pharmacokinetics within the dose range 0.5 to 2.5 mg/kg [58], we chose the pre-administration time for challenging the ligands for 5 min in the blocking studies. Haloperidol and the unlabelled SIG343/SIG353 were able to block the uptake of the radiotracers in organs of high concentrations of σ receptors in naïve animals (data not shown), especially in the case of ^18^F-SIG343. This indicates the concentration and the pre-administration time parameters of the competitive ligands used in these experiments seemed to be appropriate. However, the significant blocking of radiotracer uptake by competing ligands was not observed in the tumour for both radiotracers in the current study. The discrepancy in the results of *in vitro* cell uptake inhibition studies (*in vitro* specificity) and those of blocking studies (*in vivo* specificity) indicated that the pre-administration time of 5 min for these challenging ligands used in the current study may not be sufficient for the drugs exerting their pharmacological action. This is highly likely because in an *in vitro* system, the drugs have free access to cell membrane receptors and are only limited by their affinity, appropriate concentration used and the intrinsic efficacy. However, in an *in vivo* environment, in addition to those parameters mentioned above, the drugs are also limited by their pharmacokinetic properties before they can reach the target for receptor occupancy or saturation. In addition, the non-blocking of the competing drugs observed in the tumour in the present study could also be due to a number of variants such as the non-specific binding of the tracers in the tumour, internalisation or internal localisation of surface receptor-ligand complex in the tumour that was not readily blocked by the competing σ ligands when the radiotracers were administered. Other possibilities are pathophysiological status of the tumour in the animal model used including hypoxic fraction, proliferative or vasculature structure of the tumour, or even the pathophysiology of the diseased animals where the pharmacokinetics of both competing drug and radiotracer could also be compromised. Taken together, these factors could influence the absorption and distribution of competing drugs. Therefore, while competing σ ligands showed high binding affinity in the *in vitro* assay system, their pharmacokinetics may not be able to reach a sufficiently high concentration, or at a sufficient time, at the binding sites in solid tumours to exert their competing effects in our *in vivo* biodistribution study.

High amount of intact tracer in the tumour is desirable as it represents the stability of the radiotracer in the target. Intact ^18^F-SIG343 and ^18^F-SIG353 were 80% and 65% in the tumour, respectively at 120 min, indicating a moderate *in vivo* stability.

Unlike the biodistribution, PET imaging of ^18^F-SIG343 and ^18^F-SIG353 showed lower tumour uptake (0.9%ID/cc and 1.3%ID/cc, respectively), resulting in low tumour-to-background contrast which hampered the potential of the radiotracers as tumour markers. PET images showed high radioactive uptake in the abdominal cavity; hence, high background activity which is consistent with the biodistribution data where high uptake in the liver, spleen, lung, heart, pancreas and kidney was observed. The discrepancy in the results between *in vivo* PET and *ex vivo* biodistribution studies may be attributed to several reasons, including methodological differences in the techniques employed or method quantification/analysis of the images.

Firstly, methodological issues of data analysis should be considered. The percentage of injected dose (%ID/g) which is conventionally used in *in vivo* studies gives an absolute index in binding which means that it reflects specific and non-specific binding in targets (i.e. tumour in this animal model), radioligand present in the systemic blood circulation and possibly radioactive metabolites in the tumour and blood circulation [59], as the metabolisms of ^18^F-SIG343 and ^18^F-SIG353 have not been fully assessed. However, results of the metabolite studies indicated that the intact radiotracers were 65% to 80% in the tumour; therefore, the fate of the remaining fraction of the radiotracers cannot be fully concluded until the metabolites are identified and quantified. The second factor is the potential effect of inhalation anaesthesia used in *in vivo* studies, but not in *ex vivo* studies, which may also interfere with the kinetics of the radiotracer, especially in terms of tissue equilibrium and the extent of tissue perfusion [60]. Finally, another factor is the difference in concentrations of the radioligand used, for instance, 1 MBq in *ex vivo* studies compared with approximately 5 to 16 MBq in PET studies. High radioactivity retention in the tail (approximately 20%ID/cc) observed for both radiotracers indicated a misinjection possibility which could result in less radiotracers available for the systemic circulation, hence low radiotracer uptake in the target. Taken together, these factors could contribute to the low uptake of the radiotracers in the tumour and to the discrepancy in results between *ex vivo* and *in vivo* studies.

Future studies using longer scans (>120 min) and longer half-life radioisotopes compared to ^18^F and considering that the amount of radiotracers in the tumour remained constant, the issue of low tumour-to-background contrast could be resolved.

## Conclusions

The σ_2_ receptor is overexpressed in a variety of human tumour cell lines and is a biomarker for tumour cell proliferation, making it an attractive target for the development of new radiotracers for tumour detection and assessment of proliferative status using PET and SPECT imaging. Two lead compounds from a series of novel phthalimido compounds, SIG343 and SIG353, showing high affinity and selectivity for the σ_2_ receptor *in vitro* were selected for further development of the ^18^F-labelled PET tracer. ^18^F-SIG343 and ^18^F-SIG353 were evaluated in tumour-bearing mice, and their potential to image the σ_2_ receptor *in vivo* was investigated. Biodistribution studies showed high uptake and suitable kinetics of both radiotracers in organs known to express σ receptors. Relatively high tumour-to-blood and tumour-to-muscle uptake ratios were observed for both ^18^F-SIG343 and ^18^F-SIG353. Furthermore, we were able to demonstrate that ^18^F-SIG343 and ^18^F-SIG353 remained moderately stable *in vivo*. However, blocking studies could not fully confirm that the uptake of ^18^F-SIG343 and ^18^F-SIG353 in the tumour was σ_2_-receptor-specific. PET images showed low tumour-to-background contrast and high radioactivity localisation in the abdominal cavity for both radiotracers making them less ideal candidates for PET tumour imaging in the current animal model. Continuing the work in this direction, full potential of these radiotracers will be revisited in more appropriate models using different designed PET/SPECT protocols and suitable radioisotopes of longer half-lives to address the current low tumour-to-background contrast issue.

## Competing interests

The authors declare that they have no competing interests.

## Authors’ contributions

VN carried out radioreceptor binding studies, analysis and interpretation of data, performed statistical analysis, and drafted the manuscript. TP synthesised and radiosynthesised the compounds, carried out metabolite studies and revised the manuscript. CF synthesised the compounds. PB, ED, GR and NH participated in the biodistribution and blocking studies. IG developed the HPLC metabolite study's method. AA analysed the PET data. FM wrote the animal ethics protocols and participated in the biodistribution and blocking studies. MG supervised the research group. AK contributed to conception of the study. RS carried out the *in vitro* and *in vivo* studies, participated in analysis and interpretation of data, and revised the manuscript. All authors read and approved the final manuscript.

## Authors’ information

VN is a BPharm (University of South Australia, Australia) and PhD (Pharmacology, University of Sydney, Australia) graduate. He is holding the current position as radiotracer evaluation pharmacologist-in-charge. His main interests include radiotracer evaluation, drug pharmacodynamics/pharmacokinetics and G-protein signalling pathway investigations.

## References

[B1] Martin WR Eades CG Thompson JA Huppler RE Gilbert PE The effects of morphine- and nalorphine-like drugs in the nondependent and morphine-dependent chronic spinal dog J Pharmacol Exp Ther 1976 3 517 532 945347

[B2] Walker JM Bowen WD Walker FO Matsumoto RR de Costa B Rice KC Sigma receptors: biology and function Pharmacol Rev 1990 3 355 402 1964225

[B3] Quirion R Bowen WD Itzhak Y Junien JL Musacchio JM Rothman RB Su TP Tam SW Taylor DP A proposal for the classification of sigma binding sites Trends Pharmacol Sci 1992 3 85 86 1315463 10.1016/0165-6147(92)90030-a

[B4] Hellewell SB Bowen WD A sigma-like binding site in rat pheochromocytoma (PC12) cells: decreased affinity for (+)-benzomorphans and lower molecular weight suggest a different sigma receptor form from that of guinea pig brain Brain Res 1990 3 244 253 10.1016/0006-8993(90)91143-5 2174717

[B5] McLean S Weber E Autoradiographic visualization of haloperidol-sensitive sigma receptors in guinea-pig brain Neuroscience 1988 3 259 269 10.1016/0306-4522(88)90024-3 2839797

[B6] Walker JM Bowen WB Roberts AH de Costa BR Rice KC van Ree JM, Mulder AH, Wiegant VM, Van Wimersma-Greidanus TB Autoradiographic distribution of [3H]-(+)-pentazocine binding sites in guinea pig brain Proceedings of the International Narcotics Research. New Leads in Opioid Research Conference 1990 Amsterdam: Excerpta Medica-Elsevier 263 264

[B7] Samovilova NN Nagornaya LV Vinogradov VA (+)-[3H]SK&F 10,047 binding sites in rat liver Eur J Pharmacol 1988 3 259 264 10.1016/0014-2999(88)90784-4 3366176

[B8] Wolfe SA Jr Culp SG de Souza EB Sigma-receptors in endocrine organs: identification, characterization, and autoradiographic localization in rat pituitary, adrenal, testis, and ovary Endocrinology 1989 3 1160 1172 10.1210/endo-124-3-1160 2537173

[B9] Dumont M Lemaire S Interaction of 1,3-di(2-[5-3H]tolyl)guanidine with σ2 binding sites in rat heart membrane preparations Eur J Pharmacol 1991 3 245 248 10.1016/0014-2999(91)90176-Q 1665796

[B10] Hellewell SB Bruce A Feinstein G Orringer J Williams W Bowen WD Rat liver and kidney contain high densities of sigma 1 and sigma 2 receptors: characterization by ligand binding and photoaffinity labeling Eur J Pharmacol 1994 3 9 18 10.1016/0922-4106(94)90115-5 7925616

[B11] Kekuda R Prasad PD Fei YJ Leibach FH Ganapathy V Cloning and functional expression of the human type 1 sigma receptor (hSigmaR1) Biochem Biophys Res Commun 1996 3 553 558 10.1006/bbrc.1996.1842 8954936

[B12] Hanner M Moebius FF Flandorfer A Knaus HG Striessnig J Kempner E Glossmann H Purification, molecular cloning, and expression of the mammalian sigma1-binding site Proc Natl Acad Sci 1996 3 8072 8077 10.1073/pnas.93.15.8072 8755605 PMC38877

[B13] Seth P Fei YJ Li HW Huang W Leibach FH Ganapathy V Cloning and functional characterization of a sigma receptor from rat brain J Neurochem 1998 3 922 931 9489711 10.1046/j.1471-4159.1998.70030922.x

[B14] Pan YX Mei J Xu J Wan BL Zuckerman A Pasternak GW Cloning and characterization of a mouse sigma1 receptor J Neurochem 1998 3 2279 2285 9603192 10.1046/j.1471-4159.1998.70062279.x

[B15] Mei J Pasternak GW Molecular cloning and pharmacological characterization of the rat sigma1 receptor Biochem Pharmacol 2001 3 349 355 10.1016/S0006-2952(01)00666-9 11434908

[B16] Hayashi T Su TP Sigma-1 receptor chaperones at the ER-mitochondrion interface regulate Ca(2+) signaling and cell survival Cell 2007 3 596 610 10.1016/j.cell.2007.08.036 17981125

[B17] Tsai SY Hayashi T Mori T Su TP Sigma-1 receptor chaperones and diseases Cent Nerv Syst Agents Med Chem 2009 3 184 189 10.2174/1871524910909030184 20021352 PMC3150837

[B18] Xu J Zeng C Chu W Pan F Rothfuss JM Zhang F Tu Z Zhou D Zeng D Vangveravong S Johnston F Spitzer D Chang KC Hotchkiss RS Hawkins WG Wheeler KT Mach RH Identification of the PGRMC1 protein complex as the putative sigma-2 receptor binding site Nat Commun 2011 3 380 21730960 10.1038/ncomms1386 PMC3624020

[B19] Ahmed IS Chamberlain C Craven RJ S2R(Pgrmc1): the cytochrome-related sigma-2 receptor that regulates lipid and drug metabolism and hormone signaling Expert Opin Drug Metab Toxicol 2012 3 361 370 10.1517/17425255.2012.658367 22292588

[B20] Su TP London ED Jaffe JH Steroid binding at sigma receptors suggests a link between endocrine, nervous, and immune systems Science 1988 3 219 221 10.1126/science.2832949 2832949

[B21] Su TP Sigma receptors. Putative links between nervous, endocrine and immune systems Eur J Biochem 1991 3 633 642 10.1111/j.1432-1033.1991.tb16226.x 1655424

[B22] McCann DJ Su TP Haloperidol-sensitive (+)[3H]SKF-10,047 binding sites (sigma sites) exhibit a unique distribution in rat brain subcellular fractions Eur J Pharmacol 1990 3 211 218 10.1016/0922-4106(90)90004-H 2163873

[B23] Klein M Canoll PD Musacchio JM SKF 525-A and cytochrome P-450 ligands inhibit with high affinity the binding of [3H]dextromethorphan and sigma ligands to guinea pig brain Life Sci 1991 3 543 550 10.1016/0024-3205(91)90469-R 1846936

[B24] Knight AR Noble A Wong EHF Middlemiss DN The subcellular distribution and pharmacology of the sigma recognition site in the guinea-pig brain and liver Mol Neuropharmacol 1991 3 71 75

[B25] Ross SB Heterogeneous binding of sigma radioligands in the rat brain and liver: possible relationship to subforms of cytochrome P-450 Pharmacol Toxicol 1991 3 293 301 10.1111/j.1600-0773.1991.tb01242.x 1650944

[B26] Huang Y-S Lu H-L Zhang L-J Wu Z Sigma-2 receptor ligands and their perspectives in cancer diagnosis and therapy Med Res Rev 2013 3 1 35 10.1002/med.21297 23922215

[B27] Vilner BJ John CS Bowen WD Sigma-1 and sigma-2 receptors are expressed in a wide variety of human and rodent tumor cell lines Cancer Res 1995 3 408 413 7812973

[B28] John CS Bowen WD Varma VM McAfee JG Moody TW Sigma receptors are expressed in human non-small cell lung carcinoma Life Sci 1995 3 2385 2392 10.1016/0024-3205(95)00232-U 7791525

[B29] Wang B Rouzier R Albarracin CT Sahin A Wagner P Yang Y Smith TL Meric-Bernstam F Marcelo Aldaz C Hortobagyi GN Pusztai L Expression of sigma 1 receptor in human breast cancer Breast Cancer Res Treat 2004 3 205 214 10.1007/s10549-004-6590-0 15528963

[B30] Aydar E Onganer P Perrett R Djamgoz MB Palmer CP The expression and functional characterization of sigma (sigma) 1 receptors in breast cancer cell lines Cancer Lett 2006 3 245 257 10.1016/j.canlet.2005.11.011 16388898

[B31] Kashiwagi H McDunn J Simon P Goedegebuure P Xu J Jones L Chang K Johnston F Trinkaus K Hotchkiss R Mach R Hawkins W Selective sigma-2 ligands preferentially bind to pancreatic adenocarcinomas: applications in diagnostic imaging and therapy Mol Cancer 2007 3 48 10.1186/1476-4598-6-48 17631687 PMC1939854

[B32] Mach RH Smith CR al-Nabulsi I Whirrett BR Childers SR Wheeler KT Sigma 2 receptors as potential biomarkers of proliferation in breast cancer Cancer Res 1997 3 156 161 8988058

[B33] Bem WT Thomas GE Mamone JY Homan SM Levy BK Johnson FE Coscia CJ Overexpression of sigma receptors in nonneural human tumors Cancer Res 1991 3 6558 6562 1660342

[B34] Vilner BJ Bowen WB Mechanism for neuromodulation and protection? Mechanism for Neuromodulation and Protection 1992 Ann Arbor: NPP Books 341 353

[B35] Al-Nabulsi I Mach RH Wang LM Wallen CA Keng PC Sten K Childers SR Wheeler KT Effect of ploidy, recruitment, environmental factors, and tamoxifen treatment on the expression of sigma-2 receptors in proliferating and quiescent tumour cells Br J Cancer 1999 3 925 933 10.1038/sj.bjc.6690789 10576647 PMC2362949

[B36] Wheeler KT Wang LM Wallen CA Childers SR Cline JM Keng PC Mach RH Sigma-2 receptors as a biomarker of proliferation in solid tumours Br J Cancer 2000 3 1223 1232 10.1054/bjoc.1999.1067 10735510 PMC2363350

[B37] Difilippantonio S Chen Y Pietas A Schluns K Pacyna-Gengelbach M Deutschmann N Padilla-Nash HM Ried T Petersen I Gene expression profiles in human non-small and small-cell lung cancers Eur J Cancer 2003 3 1936 1947 10.1016/S0959-8049(03)00419-2 12932674

[B38] Crudden G Loesel R Craven RJ Overexpression of the cytochrome p450 activator hpr6 (heme-1 domain protein/human progesterone receptor) in tumors Tumour Biol 2005 3 142 146 10.1159/000086485 15970648

[B39] van Waarde A Rybczynska AA Ramakrishnan N Ishiwata K Elsinga PH Dierckx RA Sigma receptors in oncology: therapeutic and diagnostic applications of sigma ligands Curr Pharm Des 2010 3 3519 3537 10.2174/138161210793563365 21050178

[B40] Hou C Tu Z Mach R Kung HF Kung MP Characterization of a novel iodinated sigma-2 receptor ligand as a cell proliferation marker Nucl Med Biol 2006 3 203 209 10.1016/j.nucmedbio.2005.10.001 16546674

[B41] Rowland DJ Tu Z Xu J Ponde D Mach RH Welch MJ Synthesis and in vivo evaluation of 2 high-affinity 76Br-labeled sigma2-receptor ligands J Nucl Med 2006 3 1041 1048 16741315

[B42] Tu Z Dence CS Ponde DE Jones L Wheeler KT Welch MJ Mach RH Carbon-11 labeled sigma2 receptor ligands for imaging breast cancer Nucl Med Biol 2005 3 423 430 10.1016/j.nucmedbio.2005.03.008 15982571

[B43] Tu Z Xu J Jones LA Li S Dumstorff C Vangveravong S Chen DL Wheeler KT Welch MJ Mach RH Fluorine-18-labeled benzamide analogues for imaging the sigma2 receptor status of solid tumors with positron emission tomography J Med Chem 2007 3 3194 3204 10.1021/jm0614883 17579383

[B44] Tu Z Xu J Jones LA Li S Zeng D Kung MP Kung HF Mach RH Radiosynthesis and biological evaluation of a promising sigma(2)-receptor ligand radiolabeled with fluorine-18 or iodine-125 as a PET/SPECT probe for imaging breast cancer Appl Radiat Isot 2010 3 2268 2273 10.1016/j.apradiso.2010.06.004 20594864 PMC2937058

[B45] Chu W Xu J Zhou D Zhang F Jones LA Wheeler KT Mach RH New N-substituted 9-azabicyclo[3.3.1]nonan-3alpha-yl phenylcarbamate analogs as sigma2 receptor ligands: synthesis, in vitro characterization, and evaluation as PET imaging and chemosensitization agents Bioorg Med Chem 2009 3 1222 1231 10.1016/j.bmc.2008.12.025 19119012 PMC2670245

[B46] Waterhouse RN Determination of lipophilicity and its use as a predictor of blood–brain barrier penetration of molecular imaging agents Mol Imaging Biol 2003 3 376 389 10.1016/j.mibio.2003.09.014 14667492

[B47] Hajipour AR Guo LW Pal A Mavlyutov T Ruoho AE Electron-donating para-methoxy converts a benzamide-isoquinoline derivative into a highly Sigma-2 receptor selective ligand Bioorg Med Chem 2011 3 7435 7440 10.1016/j.bmc.2011.10.046 22055714 PMC3229200

[B48] DeHaven-Hudkins DL Fleissner LC Ford-Rice FY Characterization of the binding of [3H](+)-pentazocine to sigma recognition sites in guinea pig brain Eur J Pharmacol 1992 3 371 378 10.1016/0922-4106(92)90153-M 1359973

[B49] DeHaven-Hudkins DL Ford-Rice FY Allen JT Hudkins RL Allosteric modulation of ligand binding to [3H](+)pentazocine-defined sigma recognition sites by phenytoin Life Sci 1993 3 41 48 10.1016/0024-3205(93)90609-7 8515681

[B50] Nguyen VH Kassiou M Johnston GA Christie MJ Comparison of binding parameters of sigma 1 and sigma 2 binding sites in rat and guinea pig brain membranes: novel subtype-selective trishomocubanes Eur J Pharmacol 1996 3 233 240 10.1016/0014-2999(96)00395-0 8891604

[B51] Cheng Y Prusoff WH Relationship between the inhibition constant (Ki) and the concentration of inhibitor which causes 50 per cent inhibition (IC50) of an enzymatic reaction Biochem Pharmacol 1973 3 3099 3108 10.1016/0006-2952(73)90196-2 4202581

[B52] Hilton J Yokoi F Dannals RF Ravert HT Szabo Z Wong DF Column-switching HPLC for the analysis of plasma in PET imaging studies Nucl Med Biol 2000 3 627 630 10.1016/S0969-8051(00)00125-6 11056380

[B53] Visser EP Disselhorst JA Brom M Laverman P Gotthardt M Oyen WJ Boerman OC Spatial resolution and sensitivity of the Inveon small-animal PET scanner J Nucl Med 2009 3 139 147 19139188 10.2967/jnumed.108.055152

[B54] Kawamura K Ishiwata K Tajima H Ishii S Matsuno K Homma Y Senda M In vivo evaluation of [(11)C]SA4503 as a PET ligand for mapping CNS sigma(1) receptors Nucl Med Biol 2000 3 255 261 10.1016/S0969-8051(00)00081-0 10832082

[B55] van Waarde A Buursma AR Hospers GA Kawamura K Kobayashi T Ishii K Oda K Ishiwata K Vaalburg W Elsinga PH Tumor imaging with 2 sigma-receptor ligands, 18 F-FE-SA5845 and 11C-SA4503: a feasibility study J Nucl Med 2004 3 1939 1945 15534066

[B56] Mach UR Hackling AE Perachon S Ferry S Wermuth CG Schwartz JC Sokoloff P Stark H Development of novel 1,2,3,4-tetrahydroisoquinoline derivatives and closely related compounds as potent and selective dopamine D3 receptor ligands Chembiochem 2004 3 508 518 10.1002/cbic.200300784 15185375

[B57] Mach RH Huang Y Freeman RA Wu L Vangveravong S Luedtke RR Conformationally-flexible benzamide analogues as dopamine D3 and sigma 2 receptor ligands Bioorg Med Chem Lett 2004 3 195 202 10.1016/j.bmcl.2003.09.083 14684327

[B58] Cheng YF Paalzow LK Linear pharmacokinetics of haloperidol in the rat Biopharm Drug Dispos 1992 3 69 76 10.1002/bdd.2510130106 1554878

[B59] Verdurand M Nguyen V Stark D Zahra D Gregoire M-C Greguric I Zavitsanou K Comparison of cannabinoid CB1 receptor binding in adolescent and adult rats: a positron emission tomography study using [18 F]MK-9470 Int J Mol Imaging 2011 3 548123 22187642 10.1155/2011/548123 PMC3236487

[B60] Stachnik J Inhaled anesthetic agents Am J Health Syst Pharm 2006 3 623 634 10.2146/ajhp050460 16554286

